# Burger Model as the Best Option for Modeling of Viscoelastic Behavior of Resists for Nanoimprint Lithography

**DOI:** 10.3390/ma14216639

**Published:** 2021-11-04

**Authors:** Hubert Grzywacz, Piotr Jenczyk, Michał Milczarek, Marcin Michałowski, Dariusz M. Jarząbek

**Affiliations:** 1 Institute of Fundamental Technological Research, Polish Academy of Sciences, Pawińskiego 5B, 02-106 Warsaw, Poland; hgrzywa@ippt.pan.pl (H.G.); pjenczyk@ippt.pan.pl (P.J.); mmilcz@ippt.pan.pl (M.M.); 2 Faculty of Mechatronics, Warsaw University of Technology, Boboli 8, 02-525 Warsaw, Poland; marcin.michalowski@pw.edu.pl

**Keywords:** PMMA, atomic force microscopy-based nanoindentation, Young’s modulus, hardness, viscosity, Burger creep model, nanoimprint lithography

## Abstract

In this study, Atomic Force Microscopy-based nanoindentation (AFM-NI) with diamond-like carbon (DLC) coated tip was used to analyze the mechanical response of poly(methyl methacrylate) (PMMA) thin films (thicknesses: 235 and 513 nm) on a silicon substrate. Then, Oliver and Pharr (OP) model was used to calculate hardness and Young’s modulus, while three different Static Linear Solid models were used to fit the creep curve and measure creep compliance, Young’s modulus, and viscosity. Values were compared with each other, and the best-suited method was suggested. The impact of four temperatures below the glass transition temperature and varied indentation depth on the mechanical properties has been analyzed. The results show high sensitivity on experiment parameters and there is a clear difference between thin and thick film. According to the requirements in the nanoimprint lithography (NIL), the ratio of hardness at demolding temperature to viscosity at molding temperature was introduced as a simple parameter for prediction of resist suitability for NIL. Finally, thinner PMMA film was tentatively attributed as more suitable for NIL.

## 1. Introduction

The progressing miniaturization of devices and structures enforces extensive research on the nano-scale behavior of materials. Such behavior can prominently differ from macro-scale and negative effects are prone to be extremely hard to overcome while adapting technologies to the ever-increasing application’s requirements. The technology inclined towards various problems in nano-scale is lithography [1].

As the manufacturing method is exceptionally important in the fabrication of micro- and nano- electromechanical systems (MEMS and NEMS), lithography offers various subtypes [2]. Among them, being cost- and time- effective nanoimprint lithography (NIL) is pronounced [3]. However, a destructive nano-scale friction and adhesion between patterned mold and deformable material is inevitable. Therefore, controlling this interface is crucial for the minimization of the defects’ generation [4].

High interfacial adhesion, and so-caused defects (fracture, deformation, delamination), is the most important factor inhibiting industrial applications of NIL.

Decreasing adhesion and understanding the interfacial phenomena are of constant research focus. Approaches to these problems include exploring new pairs of materials, mold surface modifications, and finetuning parameters of NIL [5] and new investigation methods [6].

Tackling these problems is accelerated by application of the atomic force microscopy (AFM) [7]. AFM enables relatively fast testing of interfacial properties between pairs of materials. For example, poly(ethylene terephthalate) (PET) were investigated with AFM for temperature-dependent mechanical properties [8]. Additionally, a tip of AFM cantilever can be coated and mimic mold surface modifications in NIL [9].

Coating the tip with diamond-like-carbon (DLC) was shown to enable a repeated reproduction of nanostructures, even without anti-sticking treatment [10].

Yet, the applicative potential of such procedure would be broadened by understanding the interactions between patterned mold and imprinted material. Parameters of this contact are of primary concern in NIL, including viscosity [11], creep [12], and relaxation [13].

Another issue for obtaining a feasible NIL is an unknown influence of the substrate on resist/mold interactions. Those interactions could significantly change the behavior of the resist compared to bulk during application of the heat and pressure in NIL. The transition between commercial NIL process and experimental investigation should take into account the materials’ mechanical behavior across the scales [14] as well as reliable model should be chosen. For thermal NIL, usually poly(methyl methacrylate) (PMMA) thin film is used as a resist and Si wafer as its substrate [15].

Much scientific interest in this field was put onto microscopic level. For instance, in [16], influence of the hygrothermal aging of PMMA on creep behaviors was investigated by indentation with max. force of about 20 mN and indentation depth 2000 nm. A three-order generalized Kelvin rheological model was adopted to simulate the creep responses. In addition, nano-indentation was employed to investigate creep of PMMA—in [17] AFM and nano-indenter were used to carry out the experiments. However, for AFM the force at creep was 5 μN. In [12], creep was analyzed experimentally and numerically for tensile tests of PMMA and three constitutive models were proposed. Creep behavior was widely studied for broad spectrum of both materials and loading. For PMMA, tensile behavior was reported and modified Burger model has been proposed to increase the accuracy [12]. Nano-indentation tests were performed to study creep performance [16,18] but studied loads exceeded 1 mN.

In sub-micron scale, a few time- or temperature dependent mechanical investigations were conducted. In [19], an analytical model for prediction of mechanical response was developed. However, one of the main assumptions is that tip (hence indentation imprint) is spherical. In [20], a Kelvin model is introduced to successfully predict viscoelastic recovery of PMMA after AFM indentation at various temperatures. In [21], indentation response of 35 nm thick PMMA film is studied, however the investigation do not mention creep behavior. Other papers discussing mechanical behavior of PMMA include [21,22,23,24]. Yet, the link between those experiments and NIL is omitted. The starting point would be to choose a temperature of demolding (usually about 20 °C lower than glass transition temperature) according to viscous properties at given pattern height to resist thickness ratio. In addition, choosing the appropriate visco-elastic model is of significance.

Therefore, in this paper, PMMA-DLC mechanical interactions in nano-scale were investigated to start connecting AFM results with optimizing NIL parameters. The influence of PMMA thickness (235 and 513 nm), indentation load (200, 300, 400 and 500 nN), and environment temperature (20, 40, 60 and 80 °C) on those interactions is reported. In addition, a creep performance is studied. Oliver-Pharr approach is employed to analyze indentation curve and calculate stiffness, hardness, and Young’s modulus. Afterwards three Standard Linear Solid (SLS) models are used to calculate Young’s modulus, creep compliance and viscoelastic properties of PMMA. The comparison of Young’s moduli from these methods is shown and discussed.

## 2. Materials and Methods

In this research, two PMMA samples were prepared and then several AFM nano-indentation tests were carried out to investigate mechanical properties and creep performance. Mathematical methods (Oliver-Pharr method and Burger Creep Model) and data treatment (tip geometry analysis) are shown.

### 2.1. Sample Preparation

Samples of PMMA in the form of thin films were prepared by spin-coating. The raw material was sourced from Allresist GmbH (Strausberg, Germany) in the form of an e-beam resist AR-P 672.045. The procedure for PMMA samples preparation was presented in our earlier work [25]. In this work films with the thickness of 235 nm and 513 nm were used. Samples properties and their names used throughout this paper are summarized in Table 1.

### 2.2. AFM Tests

For nano-indentation tests, a Flex-Axiom AFM from Nanosurf (Liestal, Switzerland) was used with an additional vibration-isolation stage. The experiments were conducted in PMMA temperature of 20, 40, 60, and 80 °C, which was controlled by sample-heating stage with an accuracy of 2 °C. The relative air humidity (RH) was maintained at 25 ± 5%. NSC14/Hard/ALBS cantilever with tip coated with diamond-like-carbon (DLC) was used (Young’s modulus = 1147 GPa, Poisson’s ratio = 0.07). For all measurements, the loading rate was 40 nN/s and was equal to the unloading rate. PID parameters for feedback loop remained constant (P = 750, I = 10, D = 0). Translation of AFM parameters to indentation inputs is considered in Supplementary Materials Section 1. The use of an AFM allows measurements with lower forces and low depth indentations. During these measurements loads of 200, 300, 400, and 500 nN were applied. To investigate creep behavior, dwell time was set to 40 s.

### 2.3. Calibration

Normal force calibration was described in our earlier paper [25]. The stiffness of the cantilever was measured with the Sader method [26,27] and was equal to 3.4162 N/m with an uncertainty of 2%. Sensitivity (photodetector constant) was measured by the loading curves method [28] on sapphire substrate and was 75.5 nm/V with an uncertainty of 1.3%. Indentation depth h was calculated as a difference between the elongation of the piezo-based actuator and tip displacement measured with a four-sectional photodiode (PSD), starting at the tip-sample contact point. Afterward, elastic deformation was considered for depth of contact according to the Sneddon model. From contact depth, contact area, which is crucial for Oliver and Pharr and SLS models, was calculated as described in Supplementary Materials Section 2. The results were presented in Tables S1 and S2 and Figure S1.

### 2.4. Mathematical Methods

In this paper, we present an analysis of PMMA indentation with the Oliver-Pharr method to find hardness and Young’s modulus of samples, mathematical representation of it is described in Supplementary Materials Section 3. We also use 3 SLS models, in Maxwell, Kelvin, and Burger form to calculate Young’s modulus, creep compliance, and viscosity just as depicted in Supplementary Materials Sections 4 and 5. Schematic representations, creep compliances and reduced elastic modulus equations were presented in Table S3. During these calculations, uncertainty was calculated as the exact differential of each function. Young’s modulus obtained from both methods is compared.

## 3. Results

**Figure 1:**
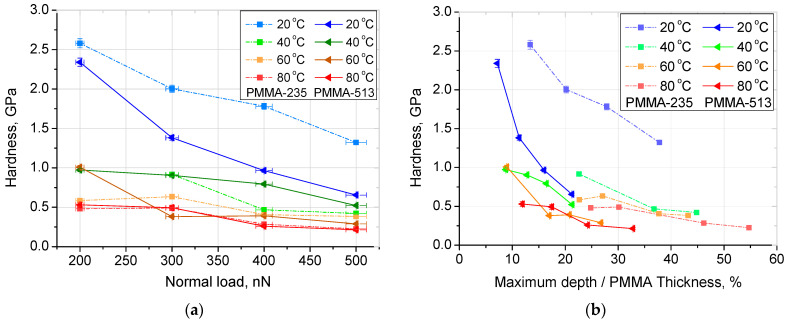
Hardness, obtained by Oliver and Pharr model: (**a**) versus maximum load for PMMA-235 and PMMA-513; (**b**) versus ratio: maximum depth nanoindentation/PMMA thickness, for PMMA-235 and PMMA-513. Lines are just guides for the eyes.

Based on the measurements carried out in this work analysis of some material properties can be accomplished. Hardness in relation to normal load, which impacts the depth of contact, temperature, and PMMA layer thickness were investigated. These are presented in Figure 1. Easily visible is the decrease of hardness with the increase of normal load, and depth of contact as well as a decrease in hardness with the increase of temperature.

**Figure 2:**
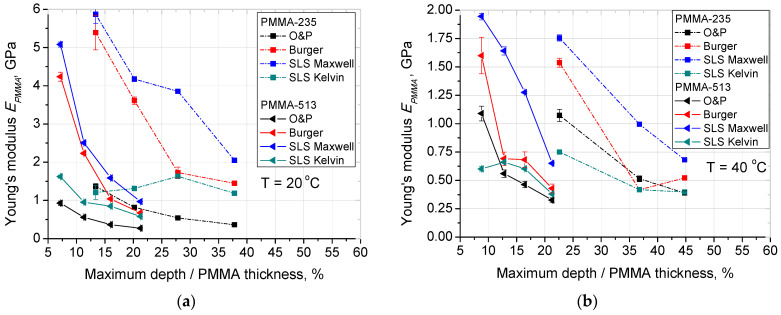

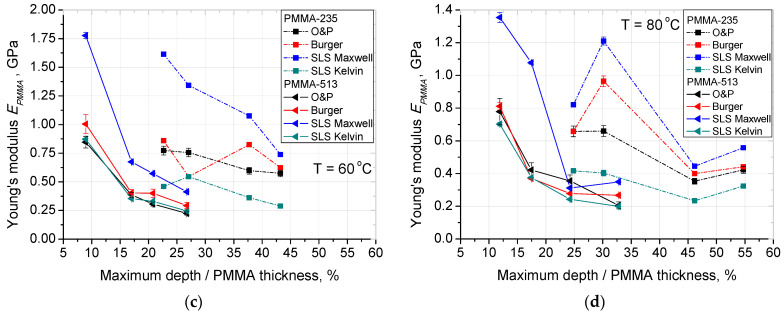
Analysis of PMMA Young’s Modulus obtained by: Oliver and Pharr, Burger model, SLS-Maxwell model and SLS-Kelvin model for PMMA thin films of 235 nm and 513 nm (**a**) modulus at 20 °C; (**b**) modulus at 40 °C; (**c**) modulus at 60 °C, (**d**) modulus at 80 °C. Lines are just guides for the eyes.

Oliver and Pharr model was also used to calculate Young’s Modulus, this property was also calculated with the use of SLS models. As a result, the modulus was calculated by four different methods for each film thickness, temperature, normal load pair. The results are shown in Figure 2. For most cases, a decrease of Young’s modulus with increased depth of indentation can be observed. As well as a decrease of Young’s modulus with the increase of temperature.

**Figure 3:**
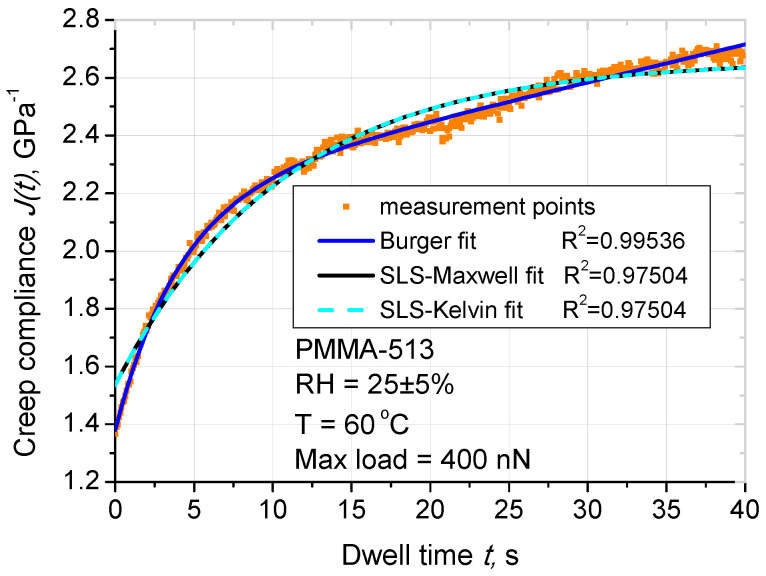
Analysis of SLS models: Burger model, SLS-Maxwell model and SLS-Kelvin model, creep compliance for PMMA-513, temperature 60 °C, maximum load 400 nN—comparison used creep models.

The use of SLS models allowed the calculation of creep compliance of the PMMA thin film. Fitting of the unloading curve was carried out for all 3 models for all temperature and normal load combinations. Below in Figure 3 we can see a sample fitting of the curves and in Table 2 we have a summary of coefficients of determination R^2^ for all combinations. Bolded numbers are used to select the model that has the best fit to particular PMMA thickness, temperature, and normal load combination.

**Figure 4:**
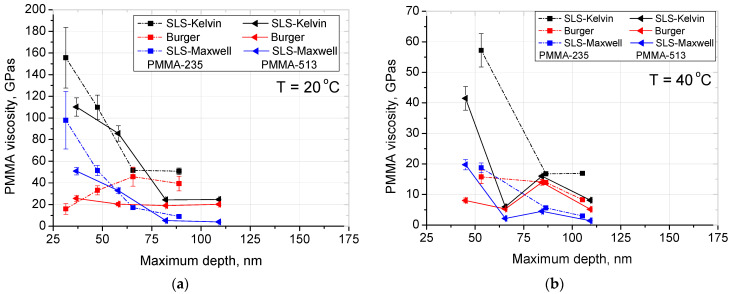

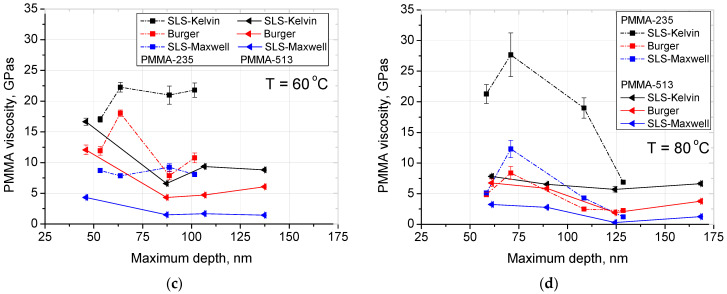
Analysis of PMMA viscosity obtained by: Burger model (η), SLS-Maxwell model (η_1_) and SLS-Kelvin model (η_1_): (**a**) viscosity at 20 °C; (**b**) viscosity at 40 °C; (**c**) viscosity at 60 °C, (**d**) viscosity at 80 °C. Lines are just guides for the eyes.

From creep compliance viscosity of the thin film was calculated for each of the models for each temperature and normal load pair. The results are shown in Figure 4. A trend of decreasing viscosity with increasing indentation depth can be seen. In addition, a decrease in viscosity with the increase of temperature is visible.

**Figure 5:**
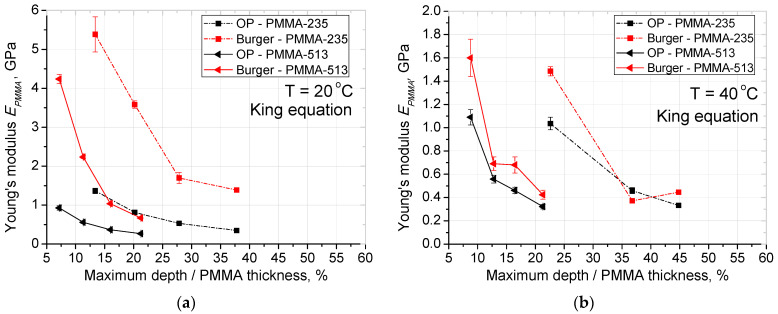

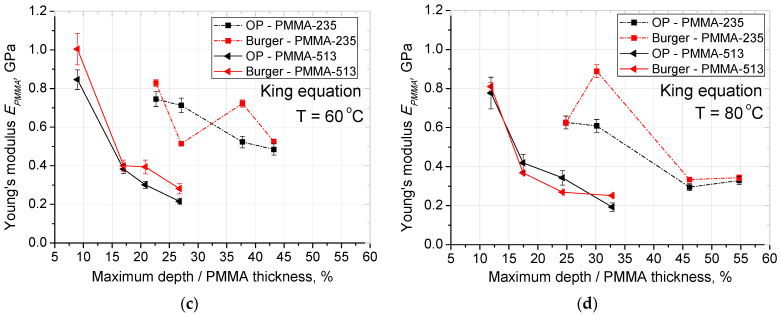
Analysis of PMMA Young’s Modulus after King’s correction for thin films; obtained by: Oliver and Pharr, Burger model, (**a**) modulus at 20 °C; (**b**) modulus at 40 °C; (**c**) modulus at 60 °C, (**d**) modulus at 80 °C. Lines are just guides for the eyes.

To take into account, the effect of the substrate, King’s method of correcting measured Young’s modulus was used in Equation (1) [29]. This was used for Oliver and Pharr (OP) calculation and the Burger model. SLS-Maxwell and SLS-Kelvin models were disregarded from further analysis due to equal or lower coefficient of determination R^2^ than the Burger model for creep compliance (see Table 2). The corrected results are shown in Figure 5, calculated according to:

(1) $$\frac{1}{E_{r}}=\frac{1-v_{film}{}^{2}}{E_{film}}\left(1-e^{-\alpha \frac{t}{a}}\right)+\frac{1-v_{substrate}{}^{2}}{E_{substrate}}e^{-\alpha \frac{t}{a}}+\frac{1-v_{indenter}{}^{2}}{E_{indenter}}$$

where *a* is the square root of contact area, *α* is a parameter dependent on contact area [29] and *t* is the thickness of the PMMA layer.

## 4. Discussion

Firstly, it should be noted that hardness shows dependence on every investigated parameter due to the fact that hardness is not an intrinsic property of the material and is usually strongly influenced by factors such as indentation depth, film thickness, samples preparation and the substrate’s material. Furthermore, the well-established Oliver-Pharr method applied also in this paper was developed for metals and it does not take the viscosity into consideration. To date there is no general agreement in the community, how to determine the hardness of thin polymer films. Hence, it should be underlined that the presented absolute values of the hardness may be significantly dependent on the applied experimental procedure and the postprocessing methodology. Nevertheless, one can compare the results and observe the clear trends in the presented results.

It was observed that the hardness decreases nearly linearly with normal load and this translates to non-linear dependence with penetration percentage. This decrease is more pronounced for lower temperatures. Similar results were previously reported by Zheng et al. [30]. Hardness also decreases at high penetration depth. Surprisingly, this decrease is observed even at depth of over 50% of PMMA thickness. This is counterintuitive, as a hard substrate should influence results and cause an increase in hardness. This leads to the conclusion that in the case of indentation of soft, viscous polymer influence of substrate is not as important as the influence of other factors. For example, an increase of hardness in low-depth indentation may be attributed to some kind of “scale effect”. In this case, it might be caused by surface tension caused by highly ordered molecules near the surface [31,32,33]. External causes may include the water adsorption layer present on the surface or error caused by insufficiently precise determination of tip area function [32].

In general, higher temperature leads to lower hardness as it increases the mobility of PMMA molecules. Additionally, 235 nm film was in general harder than 513 nm film in similar conditions. It might be attributed to the difference in the structure of PMMA caused by interactions on boundaries PMMA-substrate and PMMA-air or induced by the preparation method. The thinner film was spun at a higher speed (5400 rpm compared to 1050 rpm for thicker film) which may lead to a different arrangement of PMMA molecules.

Furthermore, Young’s modulus was calculated for 4 models. The general trend for each model remains the same. With temperature increased from 20 °C to 40 °C there is a sudden drop in Young’s modulus values and then for 60 °C and 80 °C, the decrease continues less abruptly. This indicates a threshold value of temperature which significantly increases the mobility of the polymer chains, which is in accordance with [30]. In addition, this phenomenon is more pronounced (higher relative change in Young’s modulus) for lower normal loads (and consequently lower indentation depths) what indicates that polymer molecules at the surface layer have even higher mobility. On the other hand, for 20 °C the thinner PMMA film exhibits higher Young’s modulus for corresponding normal load, which indicates the existence of sample size effect connected with tangled polymer chains and the fabrication route–it can be tentatively attributed that thinner film has a more tangled chains.

Kings [29] method of correcting Young’s modulus gives a difference that scales up with the depth of indentation in our case the tip used stays the same. After the correction the decreasing trend of Young’s modulus with increasing indentation depth is more clearly visible. The range of correction ranges from about 0% to 28% of measured value, with highest when indentation depth exceeded 50% of the film thickness for the temperature (80 °C).

As for creep compliance, for most of pairs film thickness, temperature, load Burger model presents the best fit to the creep curve. This can be seen for all temperatures and loads when analyzing PMMA 513 nm where indentation depth goes up to about 20% of the film thickness. For the thinner film, some exceptions are visible especially for higher loads. However, generally this leads us to a conclusion that Burger model is best suited to represent viscoelastic behavior of PMMA thin films. In the NIL process, plastic deformation of the resist is particularly important and it should dominate above the elastic and viscoelastic deformations. Viscoplasticity occurs in the last part of the creep curve (creep compliance increases linearly with time, see Figure 3). The Burger model consists of two dashpots: viscoelastic and (more important in NIL) viscoplastic dashpots, in contrast with the SLS-Maxwell and SLS-Kelvin models, which only consist of viscoelastic dashpot [34]. As shown in Figure 3, fit of Burger model was suitable to determine precise viscous value.

Observed decrease of viscosity with increase in temperature is in line with what was observed previously in numerous publications [35,36]. From comparing the values obtained by us to other works [17,35,36] we can see that the SLS-Kelvin model is closest to other research, although this might be caused due to similarity of the measurement method used, as the Burger model can be used to predict the behaviour during dwell with the highest accuracy. The change in viscosity with the depth of indentation can be attributed to the same change in mobility as described above when analysing Young’s modulus.

In NIL process, one needs a resist which fills perfectly the structures during molding and remains intact during and after demolding. To meet those goals, generally low viscosity during molding in elevated temperature is needed as well as high strength during demolding in lower temperature. It leads to the introduction of the ratio of the resist hardness at demolding temperature to the resist viscosity at molding temperature as a simple parameter for determination of the best NIL configuration. The higher the value, the higher the chances that a given resist is suitable for NIL process. In the scope of this paper taking 20 °C as a demolding temperature and 80 °C as a molding temperature, we obtained that this ratio is equal to 0.32 and to 0.51 for the thicker and thinner film, respectively. According to our findings, the Burger model is the best option for investigation of the viscoelastic behaviour of the NIL resists and viscosity at 80 °C was used as well as hardness from Oliver and Pharr method at 20 °C. Reported values are an average over each normal load used. However, one should take into account that AFM simulation of NIL technology is not perfect. Firstly, the stress under an AFM tip is significantly higher (a few GPa) than under a NIL mold (a few MPa). Secondly, AFM cantilever may exhibit some oscillations at higher temperature of samples due to air turbulences caused by high temperature gradient. Hence, the influence of the proposed ratio should be confirmed in the real NIL process. If confirmed then it would be a good and simple parameter for determination of the best NIL resists. The necessary experiments are going to be carried out in our further research.

## 5. Conclusions

The link between material investigations and the NIL process is not clear, despite the fact that there is a number of mechanical investigations of time- and temperature-dependent mechanical properties of polymers. Therefore, there is a need to provide an accelerated method of acquiring materials properties concerning their suitability for NIL.

In this work we analyzed four different models used for estimation of hardness, Young’s modulus, creep compliance, and viscosity of thin polymer films. Hardness measurement with the OP method allows the comparison of properties of samples measured in similar conditions. For quantitative characterization of various soft and viscous materials, the OP method should be expanded. Burgers model and OP method show similar values and trends of Young’s modulus changes with temperature and indentation depth. Burgers model gives the best fit to the creep compliance J(t) curve and was attributed as the best option for modeling viscoelastic PMMA.

In addition, in this paper the ratio of hardness at demolding temperature to viscosity at molding temperature was introduced as a simple parameter for the prediction of resist suitability for NIL. Such a parameter, obtained from an easy-and-fast AFM-NI experiment, could significantly accelerate searching for suitable deformable material for NIL. Tentatively, the thinner film was found to be better for NIL. The main future prospect is to check the effectiveness of the here-introduced suitability parameter with actual thermal NIL process. If positive, a broad scope of materials and its parameters could be investigated with relatively low effort to optimize thermal NIL.

## Figures and Tables

**Table 1:** Characterization of PMMA films.

Designation	Molecular Weight	Film Thickness, nm	Film Roughness *R_a_*, nm
PMMA-235	950 K	235 ± 5	0.328 ± 0.032
PMMA-513	950 K	513 ± 4	0.259 ± 0.033

**Table 2:** Coefficients of determination R^2^ for fittings of Burger, SLS-Maxwell and SLS-Kelvin models for experimental creep compliances curves.

Temp, °C	Maximum Load, nN	Coefficient of Determination R^2^					
		PMMA-235			PMMA-513		
		Burger	SLS-Maxwell	SLS-Kelvin	Burger	SLS-Maxwell	SLS-Kelvin
20	200	**0.91806**	0.91321	0.91321	**0.97493**	0.96742	0.96742
	300	**0.96858**	0.96360	0.96360	**0.98180**	0.97449	0.97449
	400	0.95264	**0.95271**	**0.95271**	**0.99165**	0.98766	0.98766
	500	**0.88908**	0.88654	0.88654	**0.99103**	0.98945	0.98945
40	200	-	-	-	**0.96945**	0.95529	0.95529
	300	**0.94960**	0.94450	0.94450	**0.93925**	0.93483	0.93483
	400	**0.97812**	0.97811	0.97811	**0.97910**	0.97820	0.97820
	500	**0.98132**	0.96821	0.96821	**0.96637**	0.94695	0.94695
60	200	**0.98936**	0.98654	0.98654	**0.93408**	0.92333	0.92333
	300	0.97389	**0.97394**	**0.97394**	**0.98964**	0.97310	0.97310
	400	**0.97211**	0.95928	0.95928	**0.99536**	0.97504	0.97504
	500	**0.98596**	0.98359	0.98359	**0.99524**	0.98980	0.98980
80	200	**0.94736**	0.90225	0.90225	**0.94883**	0.85429	0.85429
	300	**0.93514**	0.91228	0.91228	**0.99320**	0.99254	0.99254
	400	**0.96054**	0.93963	0.93963	**0.96540**	0.92598	0.92598
	500	0.94312	**0.97394**	**0.97394**	**0.98012**	0.96854	0.96854

## Data Availability

The datasets generated during and/or analyzed during the current study are available from the corresponding author on reasonable request.

## Supplementary Materials

The following are available online at https://www.mdpi.com/article/10.3390/ma14216639/s1, Figure S1: Investigation methodology: (**a**) 2D-map of used tip—NSC14/Hard/AL/BS, (**b**) tip area curve, (**c**) example of a registered curve during nanoindentation test, (**d**) example of a registered creep curve during dwell time, Table S1: Tip heights and tip cross areas measurement points, Table S2. Area function fit factors and values, Table S3. Rheological models for constitutive creep modeling at constant stress: J_0_, J_1_—creep compliances in GPa^−1^, τ—retardation time in s, η_0_—viscoplasticity in GPa∙s, η_1_—viscoelasticity in GPa∙s.
