# Supplementary material for: Burger Model as the Best Option for Modeling of Viscoelastic Behavior of Resists for Nanoimprint Lithography
Source: Materials (Basel). 2021 Nov 4;14(21):6639. doi: 10.3390/ma14216639 (PMC8587944; doi:10.3390/ma14216639)
Supplement: Supplementary file 1 [file materials-14-06639-s001.zip › materials-1403209-supplementary.pdf]

materials MDPI

Supplementary Materials

Burger Model as the Best Option for Modeling of Viscoelastic Behavior of Resists for Nanoimprint Lithography

Hubert Grzywacz [1,†], Piotr Jenczyk [1,†], Michał Milczarek [1], Marcin Michałowski [2], Dariusz M. Jarząbek [1,2,*]

[1] Institute of Fundamental Technological Research, Polish Academy of Sciences, Pawińskiego 5B, 02-106 Warsaw, Poland; hgrzywa@ippt.pan.pl (H.G.); pjenczyk@ippt.pan.pl (P.J.); mmilcz@ippt.pan.pl (M.M.)
[2] Faculty of Mechatronics, Warsaw University of Technology, Boboli 8, 02-525 Warsaw, Poland; marcin.michalowski@pw.edu.pl
* Correspondence: djarz@ippt.pan.pl
† The authors provided equal first-author-level contribution to this work.

Citation: Grzywacz, H.; Jenczyk, P.; Milczarek, M.; Michałowski, M.; Jarząbek, D.M. Burger Model as the Best Option for Modeling of Viscoelastic Behaviour of Resists for Nanoimprint Lithography. *Materials* **2021**, *14*, 6639. https://doi.org/10.3390/ma14216639

Academic Editor: Andrea P. Reverberi

Received: 15 September 2021
Accepted: 29 October 2021
Published: 4 November 2021

Publisher's Note: MDPI stays neutral with regard to jurisdictional claims in published maps and institutional affiliations.

Copyright: © 2021 by the author. Licensee MDPI, Basel, Switzerland. This article is an open access article distributed under the terms and conditions of the Creative Commons Attribution (CC BY) license (http://creativecommons.org/licenses/by/4.0/).

1. Nanoindentation with Atomic Force Microscope (AFM-NI)

Applied, normal force in AFM-NI method is [1]:
where: P—normal force in nN, SP—set point in V, α—cantilever sensitivity in nmV^{-1}, k—cantilever spring constant in Nm^{-1}, D—deflection, measured in PSD photodiode in nm.

Indentation depth h:

$$P = SP \cdot \alpha \cdot k = D \cdot k \quad \text{S1}$$

$$h = (Z - Z_o) - D \quad \text{S2}$$

where: Z—current vertical position of AFM piezo actuator in nm, Z_o—contact point in nm.

Loading (Unloading) Rate is equal:

$$|LR| = |UR| = v_{piezo} \cdot k \quad \text{S3}$$

where v_{piezo} is piezo actuator velocity in nms^{-1}.

2. Analysis of Tip Geometry

Tip geometry has a significant role in nanoindentation measurements and analysis. One of the methods to obtain contact area function, is approximation of a specific function to measurement points. In these investigations, a 2D-map of used tip was determined by GWYDDION software, subsequently tip horizontal cross section areas were obtained for appropriated tip heights, using graphic program GIMP (Figure S1). The results were presented in Table S1.

Table S1. Tip heights and tip cross areas measurement points.

Tip height, nm	11	12	31	41	51	61	71	81	91
Tip cross area, $(nm)^2$	18.85	71.54	159.28	283.16	433.08	632.81	855.74	1113.01	1410.48

Subsequently, measurement points presented in Table S1. were approximated by function [2]:

$$A(h) = C_0h^2 + C_1h + C_2h^{1/2} + C_3h^{1/4} + C_4h^{1/8} \quad \text{(S1)}$$

where C_0, … C_4 are fit factors, presented in Table S2. Coefficient of determination R^2 was 0.99996.

Table S2. Area function fit factors and values.

Fit factor	C_0	C_1	C_2	C_3	C_4
Value	0.17272	−0.22881	0.12311	0.12298	0.12289

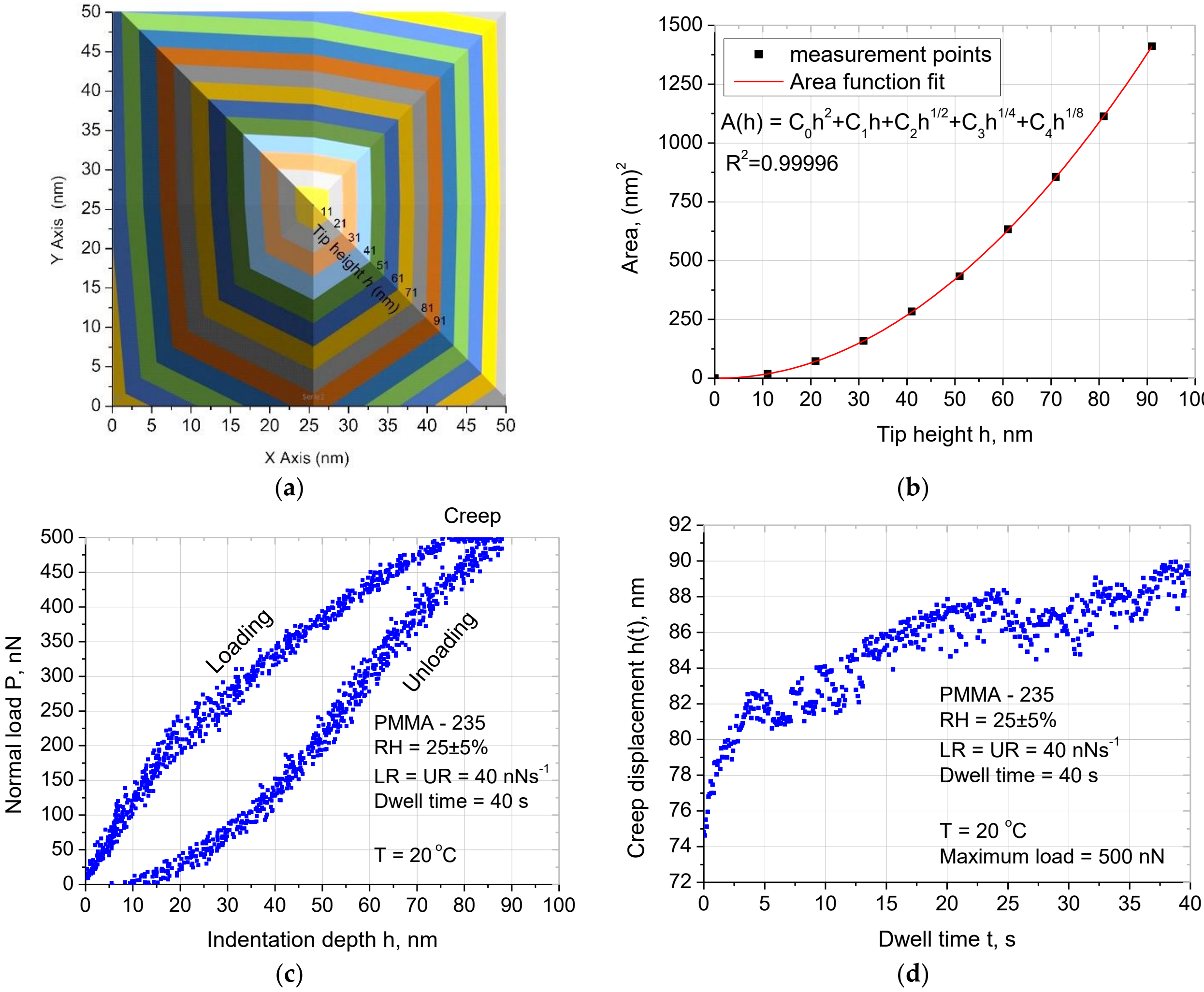

Figure S1. Investigation methodology: (**a**) 2D-map of used tip—NSC14/Hard/AL/BS, (**b**) tip area curve, (**c**) example of a registered curve during nanoindentation test, (**d**) example of a registered creep curve during dwell time.

3. Oliver and Pharr Model

To calculate samples Young's modulus E_{PMMA} and hardness H, the slope of the unloading nanoindentation curve was analyzed. Oliver & Pharr [2,3] proposed unloading fit function:

$$P = B\left(h - h_f\right)^m, \tag{S2}$$

where: *B*, h_f, m—fit factors, P—normal load in nN, h—measured nanoindentation depth in nm.

Sample stiffness *S*:

$$S = \left(\frac{\partial P}{\partial h}\right)_{h=h_{max}} = Bm\left(h_{max} - h_f\right)^{m-1}. \tag{S3}$$

where h_{max} is maximum depth nanoindentation in nm.

Feng and Ngan [4] proposed a method to correct contact stiffness, by influence of viscoelasticity. The elastic stiffness S_e is:

$$\frac{1}{S_e} = \frac{1}{S} + \frac{\dot{h}}{|UR|} \tag{S4}$$

where: S – measured stiffness (Equation (S6)) in Nm^{-1}, $\dot{h}$—creep rate just prior to unloading in nms^{-1}, $|UR| = 40\ \mathrm{nNs}^{-1}$—nanoindentation unloading rate.

The contact area is calculated from contact depth h_c:

$$h_c = h_{max} - \epsilon \frac{P_{max}}{S_e}, \tag{S5}$$

where: ϵ—constant, depend on tip geometry (for conical tip 0.72), P is maximum load in nN.

Hardness is defined by dividing the maximum *load* P_{max} by the projected contact area $A(h_c)$:

$$H = \frac{P_{max}}{A(h_c)}. \tag{S6}$$

Reduced elastic modulus E_r is depend on stiffness S_e and contact area $A(h_c)$:

$$E_r = \frac{\sqrt{\pi}}{2\beta} \cdot \frac{S_e}{\sqrt{A(h_c)}} \tag{S7}$$

where β—coefficient, depend on tip geometry; for conical tip $\beta = 1$.

PMMA Young's modulus E_{PMMA} was determined, using equation:

$$\frac{1}{E_r} = \frac{1 - v_{PMMA}^2}{E_{PMMA}} + \frac{1 - v_{DLC}^2}{E_{DLC}} \tag{S8}$$

where: $v_{PMMA} = 0.38$, $v_{DLC} = 0.07$—PMMA and DLC Poisson ratios, respectively, $E_{DLC} = 1141$ GPa—DLC Young's modulus [5].

4. Creep Compliance

Creep compliance at constant stress is dependent on tip geometry, contact area and maximum load. For conical tip [6]:

$$J(t) = \frac{2}{\pi^2\left(1 - v_{PMMA}^2\right)\operatorname{tg}\varphi} \cdot \frac{A\left(h_c(t)\right)}{P_{max}}, \tag{S9}$$

where φ is half angle of conical tip. Contact area was determined by Equation (S4), where depth $h_c(t)$ was calculated by equation:

$$h_c(t) = h_{creep}(t) - \epsilon \frac{P_{max}}{S_e}, \tag{S10}$$

where $h_{creep}(t)$ is measured creep displacement at determined P_{max}.

5. Creep Models

In Table S3. three used models of creep: SLS-Maxwell SLS-Kelvin and Burger were presented.

Table S3. Rheological models for constitutive creep modeling at constant stress: J_0, J_1—creep compliances in GPa^{-1}, τ—retardation time in s, η_0—viscoplasticity in GPa·s, η_1—viscoelasticity in GPa·s.

Creep Model	Schematic Representation	Creep Equation *J(t)*	Reduced Elastic Modulus *Er*
**SLS—Maxwell form** [7]	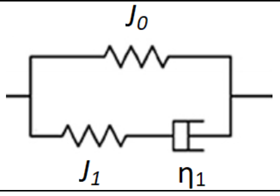	$J(t) = J_0\left(1 - \frac{J_0}{J_0 + J_1} e^{-t/\tau}\right)$<br>$\tau = \eta_1(J_0 + J_1)$	$E_r = \frac{1}{J_0} + \frac{1}{J_1}$
**SLS—Kelvin form** [8]	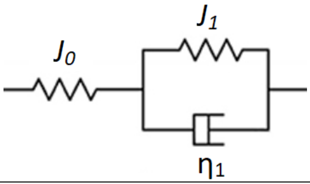	$J(t) = J_0 + J_1\left(1 - e^{-t/\tau}\right)$<br>$\tau = \eta_1 J_1$	$E_r = (J_0 + J_1)^{-1}$
**Burger** [8]	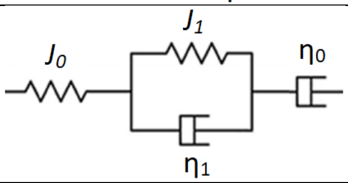	$J(t) = J_0 + J_1\left(1 - e^{-t/\tau}\right) + \frac{t}{\eta_0}$<br>$\tau = \eta_1 J_1$<br>$\eta = \left(\frac{1}{\eta_0} + \frac{1}{\eta_1}\right)^{-1}$	$E_r = (J_0 + J_1)^{-1}$

For mentioned above creep models, PMMA Young's modulus were calculated, using Equation (S11).

References

1. Suriano, R.; Credi, C.; Levi, M.; Turri, S. AFM nanoscale indentation in air of polymeric and hybrid materials with highly different stiffness. *Appl. Surf. Sci.* **2014**, *311*, 558–566, doi:10.1016/j.apsusc.2014.05.108.
2. Oliver, W.; Pharr, G. Measurement of hardness and elastic modulus by instrumented indentation: Advances in understanding and refinements to methodology. *J. Mater. Res.* **2004**, *19*, 3–20, doi:10.1557/jmr.2004.19.1.3.
3. Oliver, W.; Pharr, G. An improved technique for determining hardness and elastic modulus using load and displacement sensing indentation experiments. *J. Mater. Res.* **1992**, *7*, 1564–1583, doi:10.1557/jmr.1992.1564.
4. Tang, B.; Ngan, A.H.W. Accurate measurement of tip–sample contact size during nanoindentation of viscoelastic materials. *J. Mater. Res.* **2003**, *18*, 1141–1148, doi:10.1557/jmr.2003.0156.
5. Grzywacz, H.; Milczarek, M.; Jenczyk, P.; Dera, W.; Michałowski, M.; Jarząbek, D.M. Quantitative measurement of nanofriction between PMMA thin films and various AFM probes. *Meas.* **2021**, *168*, 108267, doi:10.1016/j.measurement.2020.108267.
6. Peng, G.; Zhang, T.; Feng, Y.; Yang, R. Determination of shear creep compliance of linear viscoelastic solids by instrumented indentation when the contact area has a single maximum. *J. Mater. Res.* **2012**, *27*, 1565–1572, doi:10.1557/jmr.2012.120.
7. A Bonfanti, A.; Kaplan, J.L.; Charras, G.; Kabla, A.J. Fractional viscoelastic models for power-law materials. *Soft Matter* **2020**, *16*, 6002–6020, doi:10.1039/d0sm00354a.
8. Dogan, M.; Kayacier, A.; Toker, Ömer S.; Yilmaz, M.T.; Karaman, S. Steady, Dynamic, Creep, and Recovery Analysis of Ice Cream Mixes Added with Different Concentrations of Xanthan Gum. *Food Bioprocess Technol.* **2013**, *6*, 1420–1433, doi:10.1007/s11947-012-0872-z.
